# Intranasal Vaccination with an Engineered Influenza Virus Expressing the Receptor Binding Subdomain of Botulinum Neurotoxin Provides Protective Immunity Against Botulism and Influenza

**DOI:** 10.3389/fimmu.2015.00170

**Published:** 2015-04-21

**Authors:** Junwei Li, Diana Diaz-Arévalo, Yanping Chen, Mingtao Zeng

**Affiliations:** ^1^Department of Biomedical Sciences, Center of Excellence for Infectious Diseases, Paul L. Foster School of Medicine, Texas Tech University Health Sciences Center, El Paso, TX, USA

**Keywords:** botulinum neurotoxin, influenza, intranasal vaccination, protective immunity, recombinant influenza vector

## Abstract

Influenza virus is a negative segmented RNA virus without DNA intermediate. This makes it safer as a vaccine delivery vector than most DNA viruses that have potential to integrate their genetic elements into host genomes. In this study, we developed a universal influenza viral vector, expressing the receptor binding subdomain of botulinum neurotoxin A (BoNT/A). We tested the growth characters of the engineered influenza virus in chicken eggs and Madin–Darby canine kidney epithelial cells (MDCK), and showed that it can be produced to a titer of 5 × 10^6^ plaque forming unites/ml in chicken eggs and MDCK cells. Subsequently, mice intranasally vaccinated with the engineered influenza virus conferred protection against challenge with lethal doses of active BoNT/A toxin and influenza virus. Our results demonstrated the feasibility to develop a dual purpose nasal vaccine against both botulism and influenza.

## Introduction

Influenza A virus is a member of family orthomyxoviridae, which contains a segmented RNA genome. With the elucidation of replication mechanism of influenza virus, generation of influenza virus from DNA became feasible and extended the research of influenza dramatically. The most important application of this system is creation of live-attenuated vaccine or the generation of influenza vector to express foreign antigens (1–3). Engineered influenza viruses expressing foreign antigens have successfully induced a vigorous immune response in mice by the intranasal immunization (4–6). In early stage of influenza virus vector development, influenza virus non-structural gene (NS) segment was engineered to express foreign antigen with deletion of the nuclear export protein (NEP), formally referred to as NS2 (7–11). With the finding of 2A cleavage sequence of picornavirus, NS gene segment was modified with multi-cistronic sites. It enlarged the containment of foreign gene, kept a high growth of engineered influenza virus, and enhanced genetic stability (7, 12).

Botulinum neurotoxins (BoNTs, A–H) are the most poisonous substances known in nature. They may be used as bioterrorism agents or in biological warfare. Currently, there is no FDA licensed botulism vaccine available for public use. Natural BoNT is divided into a light chain (L) and a heavy chain (H) (13). L chain is a globular protein with Zn^+^-metalloprotease activity (14). H chain is divided into N-terminal translocation domain and C-terminal receptor binding domain (H_C_50), which consists of N-terminus without assigned function and C-terminus with receptor binding subdomain (rbsd) (15, 16). Previously, we developed an effective adenovirus-vectored vaccine expressing H_C_50 (17). In this research, we created a recombinant influenza virus vector with a 2A cleavage site on NA. Subsequently, we generated an engineered influenza virus based on H1N1 PR8 virus, expressing the truncated H_C_50-rbsd from BoNT/A. Intranasal vaccination with this engineered influenza virus was evaluated for protection against lethal challenges with BoNT/A and influenza virus.

## Materials and Methods

### Cell culture and animal studies

Madin–Darby canine kidney epithelial cells (MDCK) were cultured in modified Eagle’s medium with 10% fetal calf serum. 293T cells were cultured in Dulbecco’s modified eagle’s medium (DMEM) with 10% fetal calf serum. BALB/c mice were purchased from the Jackson Laboratories (Bar Harbor, ME, USA). Animal experimental protocol (protocol number is 10020) was approved by the Institutional Animal Care and Use Committee at Texas Tech University Health Sciences Center, and carried out in accordance with the US Public Health Service Guide for the Care and Use of Laboratory Animals (NRC Publication, 8th ed.) and other related federal statutes and regulations of the Animal Welfare Act. MLD_50_ were determined by Reed–Muench method (18).

### Generation of engineered influenza viruses

**Figure 1:**
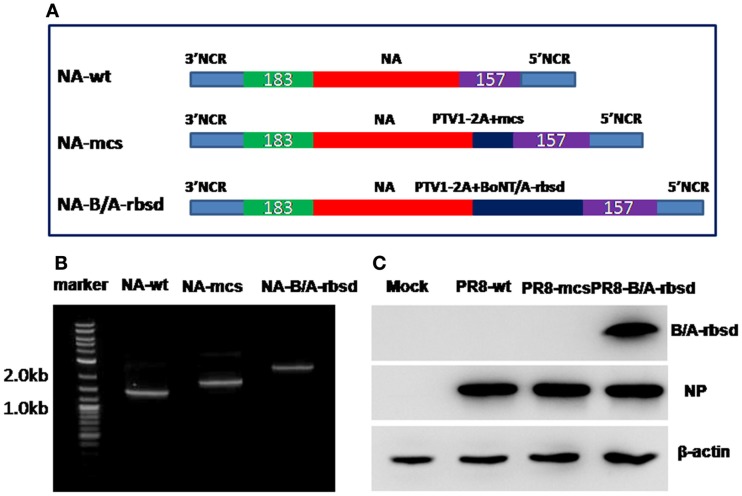
**Design and identification of influenza virus expression of BoNT/A-rbsd**. **(A)** (Top) Organization of the original (wt) NA gene segment. (Middle) Organization of the modified NA gene segment engineered with porcine teschovirus (PTV)-1 2A cleavage site and multiple cloning site (mcs). (Bottom) Organization of the modified NA gene segment engineered with receptor binding subdomain of BoNT/A toxin. **(B)** NA gene segment amplified by RT-PCR. **(C)** Receptor binding subdomain of BoNT/A toxin is expressed in MDCK cells infected with engineered influenza virus PR8-B/A-rbsd, as determined by Western blot.

First, we synthesized a gene segment coding 2A peptide followed by a multiple cloning site (mcs) (19). Then, it was linked with 185 bp of C terminal for packaging signals of NA gene segment by overlap PCR. Subsequently, the N-terminal non-coding region with ORF of NA gene segment was linked with the above gene segment by overlap PCR (Figure 1A). Finally, full-length PCR product was inserted into pHW-2000. New plasmid was designated pHW-NA-mcs. Synthesized human-codon-optimized gene segment of BoNT/A-H_C_50-rbsd (amino acid 1088-1293, GenBank: CAL82360.1), was inserted into pHW-NA-mcs, and the plasmid was named pHW-NA-B/A-rbsd. Engineered influenza viruses using PR8 influenza virus as backbone were rescued by reverse genetics with eight plasmids (20). The wild type control A/Puerto Rico/8/1934(H1N1) influenza virus (PR8) was produced by similar method.

### Viral growth and titration

About 100 pfu viruses were inoculated into allantoic cavity of a 9-day-old specific pathogen free (SPF) embryonated eggs (Charles River, CT, USA). At 72 h post-inoculation, viruses were harvested and titrated on monolayer MDCK cells by using the standard plaque assay method. To measure virus growth curve, monolayer MDCK cells were initially infected with virus at multiplicity of infection (MOI) of 0.001. After 1 h incubation, virus was removed and MDCK cells were washed with PBS supplemented with Ca^++^ and Mg^++^, then medium were replaced with fresh medium supplemented with 0.2% BSA and 1 μg/ml Tosyl phenylalanyl chloromethyl ketone (TPCK)-trypsin. At 24, 36, 48, 60, and 72 h post-infection, virus samples were collected, respectively, and titrated on monolayer MDCK cells in triplicate.

### RT-PCR

Viral RNA was extracted from virus samples using RNeasy kit (Qiagen, CA, USA), and subjected to the RT-PCR amplification by using SuperScript III Reverse Transcriptase kit (Invitrogen, CA, USA). RT-PCR products were analyzed by electrophoresis in 0.8% agarose gel. The images were taken by VersaDoc imager (BioRad, CA, USA).

### Western-blotting

Madin–Darby canine kidney epithelial cells were infected with influenza virus at MOI of 1. At 8 h post-infection, cells were harvested and lysed. Proteins were separated on 10% SDS-PAGE and transferred to nitrocellulose membrane. Then, membrane was incubated with anti-BoNT/A-H_C_50 serum (prepared in our lab), rabbit anti-NP polyclonal antibody (Immune-tech), and β-actin antibody, respectively. After being washed, membrane was incubated with goat anti-rabbit or -mice IgG-HRP for 1 h. Finally, membrane was developed with chemiluminescence HRP substrate (Takara Bio Inc.), and images were taken by using ImageQuant Las4000 (GE Health, PA, USA).

### Animal experiments

Forty-eight (48) 6-week female BALB/c mice were divided to 8 groups. Subsequently, the mice were intranasally vaccinated with 5 pfu influenza virus. At 4 weeks post-infection, mice were boosted with the same dose. Blood samples were collected on the days of immunization and challenge. On day 42, mice were challenged with 10× MLD_50_ BoNT/A toxin (BEI Resources, cat# NR-4529, NIAID, NIH) or 100× MLD_50_ PR8 influenza virus. After being challenged, mice were observed every day. In the BoNT/A toxin challenged groups, data of survived mice and dead mice were calculated. In the PR8 challenged groups, mouse weights were recorded every day. When the mouse weight loss was >25%, the mouse was euthanized and sacrificed according the guide of the Institutional Animal Care and Use Committee at Texas Tech University Health Sciences Center.

### ELISA

Briefly, microplates were coated with recombinant BoNT/A receptor binding subdomain overnight. After antigen was removed and plates were blocked, sera were diluted and added into each wells, and incubated at 4^o^C overnight. One hundred microliters of diluted goat anti-mouse IgG-Fc antibody conjugated to alkaline-phosphtase were added into each well and incubated for 1 h. Subsequently, plates were washed and pNPP substrate was added and incubated for 20 min. After sufficient color development, stop solution was added into each well. Finally, the plates were read in the Gene5 microplate reader (BioTek).

### Statistical analysis

Comparison between virus titers was performed by using *t* test calculator (http://www.graphpad.com/quickcalcs/ttest1.cfm). *P* values <0.05 were considered to be significant difference. Comparisons between vaccinated groups were performed by using a non-parametric one-ways ANOVA with the Tukey multiple comparison test and Fisher’s exact, and survival dates were analyzed by using the log-rank test. The analyses were performed by using GraphPad Prism version 5.0 for Windows (GrahPad Software). *P* values <0.05 were considered to be significant difference.

## Results

### Rescue of engineered influenza viruses

At first, NA gene segment was engineered by inserting a multi-cloning site with a 2A cleavage site. Then a universal replication-competent influenza viral vector was rescued. To demonstrate the use of this universal influenza viral vector, a 618 bp gene segment of BoNT/A H_C_50-rbsd was inserted into engineered NA gene segment. An engineered influenza virus carrying target gene segment was rescued, purified, and propagated in embryonated chicken eggs. The rescued viruses were named PR8-wt, PR8-mcs, PR8-B/A-rbsd, respectively. Then, viruses propagated in embryonated chicken eggs were harvested, aliquoted, and stored at −80°C for subsequent use.

### Confirmation of recombinant influenza virus

To confirm rescued influenza viruses carrying anticipated NA gene segment, viral RNA was extracted, and NA gene segments were amplified. Results in Figure 1B showed wild type NA gene or engineered NA gene segments were enclosed in the rescued influenza virus. Furthermore, expression of BoNT/A receptor binding subdomain was confirmed by western-blotting. Figure 1C shows the correct size of BoNT/A H_C_50-rbsd is around 26 kDa.

### Growth characteristics of engineered influenza viruses

**Figure 2:**
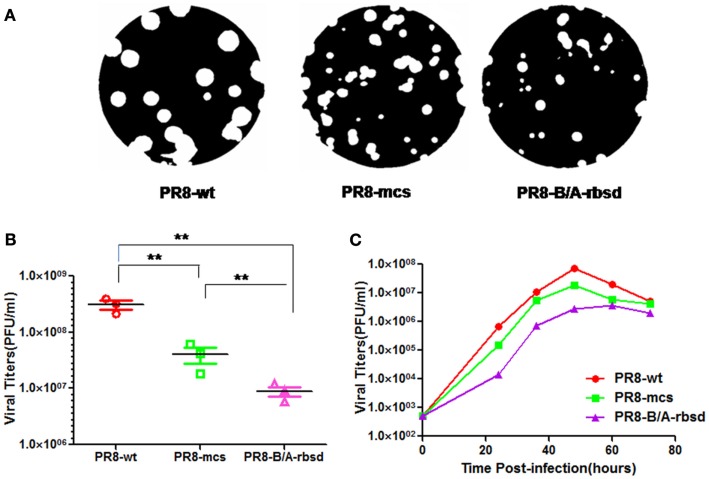
**Growth characters of wild type and engineered PR8 influenza viruses**. **(A)** Plaque formed by infection with wild type and engineered PR8 influenza viruses on monolayer MDCK cells. **(B)** Virus growth character in embryonated chicken eggs (**P < 0.05). **(C)** Virus growth curves on monolayer MDCK cells.

Plaque assay on MDCK cells was performed to analyze the growth characteristics of engineered influenza viruses. In Figure 2A, plaques formed by engineered influenza virus are smaller than that formed by wild type PR8. Furthermore, the growth characters were tested in MDCK cells and eggs. The results in Figures 2B,C showed that titers of engineered influenza viruses are lower than those of wild type. It suggested that engineering NA gene was inhibitory to replication of influenza virus. However, the engineered influenza virus propagated with reasonable titers in MDCK cells and embryonated chicken eggs. The titers could reach to 5 × 10^6^ pfu/ml.

### Humoral immune response and protection stimulated by engineered influenza virus

**Figure 3:**
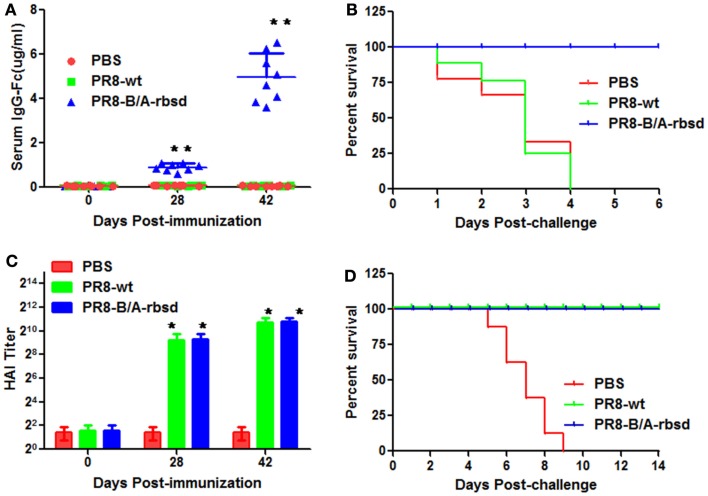
**Intranasal vaccination with the engineered influenza virus expressing BoNT/A H_C_50-rbsd conferred protection against BoNT/A and influenza virus infection**. Mice were intranasally inoculated with 5 pfu of PR8-B/A-rbsd virus at weeks 0 and 4, and challenged with 10× MLD_50_ BoNT/A toxin or 100× MLD_50_ PR8 influenza virus on day 42. **(A)** Vaccination with PR8-B/A-rbsd virus-induced antibody response against receptor binding subdomain of BoNT/A toxin (**P < 0.05 compared with PBS group or PR8-wt group). **(B)** Vaccination with PR8-B/A-rbsd conferred protection against lethal challenge with BoNT/A toxin. **(C)** Vaccination elicited antibody response against HA of PR8 influenza virus (*P > 0.05 compared with PR8-wt group). **(D)** Vaccination provided protection against lethal infection with PR influenza viruses (*n* = 8 for each group).

In Figure 3A, results showed that intranasal vaccination with the recombinant influenza virus PR8-B/A-rbsd stimulated significant humoral immune response against BoNT/A toxin receptor binding subdomain compared with wild type influenza virus PR8. In Figure 3B, results showed that vaccination with PR8-B/A-rbsd provided protection against challenge with 10× MLD_50_ active BoNT/A toxin. We also tested whether immunization with PR8-B/A-rbsd induced adaptive immunity against influenza virus infection. We tested the humoral immune response against influenza virus by using hemagglutination inhibition (HAI) method and challenged immunized mice with 100× MLD_50_ PR8 influenza virus. In Figures 3C,D, the results showed vaccination with PR8-B/A-srbd stimulated potent humoral immune response, and resulted in high HAI titers and protection against challenge with lethal PR8 influenza virus.

## Discussion

Influenza virus has plenty of advantages that make it worthy of consideration for use as a viral vector for pathogens that are problematic to vaccine development. There are well-established protocols for large-scale production of both live and inactivated influenza viruses, and live influenza vaccines have shown to elicit strong T cell immune response to stimulate mucosal and systemic responses (21, 22). Live-attenuated influenza virus may be the suitable virus vector to express foreign antigen as dual purpose vaccine. As depicted previously, there are several publications showed influenza virus could engineered to express foreign antigen; however, there is no research using influenza virus as vector to express antigen of botulism. In this research, we used mouse-adapted influenza virus as vector to express the receptor binding subdomain of BoNT/A toxin. In animal experiments, the engineered influenza viruses still have certain degree of pathogenicity in mouse (MLD_50_ is 5,000 pfu, data not shown). In the future, we will use cold-adapted influenza viruses as vectors, because research has shown cold-adapted influenza virus is much safer than seasonal one (8, 23).

Botulinum neurotoxins are the most poisonous substances in the nature and potential bioterrorism agents. With the advantage of molecular technology, identification of non-toxic domains of BoNT toxin provides a useful method to develop a promising vaccine for botulism. In this research, our results showed that the smaller receptor binding subdomain of BoNT/A toxin is an effective antigen to stimulate humoral immune response. Additional research in this direction may lead to a multivalent vaccine against all types of BoNTs using the smaller antigen (H_C_50-rbsd) instead of H_C_50 (17) from each serotype. Data from this research demonstrated the possibility to develop a dual protective vaccine against both botulism and influenza. In this research, PR8 influenza virus is an old laboratory-adapted influenza virus. Maybe it is not suitable to be as human influenza vaccine. It just provides a research platform. In further research, cold-adapted or attenuated seasonal or pandemic influenza viruses will be recruited to provide a viable vaccine for bio-defense against BoNTs and for public health emergency against potential pandemic influenza.

## Author Contributions

JL and MZ designed the research, analyzed the data, and wrote the manuscript. JL, DD-A, and YC performed experiments.

## Conflict of Interest Statement

The authors declare that the research was conducted in the absence of any commercial or financial relationships that could be construed as a potential conflict of interest.
